# Editorial for the Special Issue on Nanomaterials Photonics

**DOI:** 10.3390/mi15101268

**Published:** 2024-10-18

**Authors:** Raul Rangel-Rojo, Luciana R. P. Kassab, Tiziana Cesca

**Affiliations:** 1 Department of Optics, Centro de Investigación Científica y Educación Superior de Ensenada (CICESE), Ensenada 22880, Mexico; 2 Departamento de Ensino Geral, Faculdade de Tecnologia de São Paulo, São Paulo 01124-060, SP, Brazil; kassablm@osite.com.br; 3 NanoStructures Group (NSG), Department of Physics and Astronomy, University of Padova, Via Marzolo 8, I-35131 Padova, Italy; tiziana.cesca@unipd.it

Back in 1959, Richard Feynman, in his famous lecture, stated that “*There is plenty of room at the bottom*”, which made us aware of the possibilities of nanotechnology, i.e., the possibility to build devices with dimensions at the scale of a few nanometers, and he prompted a surge in the study of nanoscale materials [1]. These developments were delayed for many more years until the techniques necessary to build such structures as well as the techniques required for visualizing them with sufficient spatial resolution, were developed. These efforts have enabled visualizing single atoms [2] and positioning them in desired patterns, such as the famous ‘quantum corral’ [3]. Moreover, in 2016, a group of researchers at TU Delft and INL reported the storage of a paragraph of Feynman’s talk using binary code where every bit was made with a single atomic vacancy [4]. Using a scanning tunnelling microscope to manipulate thousands of atoms, the researchers wrote the text.

Originally, Feynman imagined the fabrication of miniature electromechanic devices that could perform different tasks and be capable of replicating themselves. However, the field of nanostructured materials has grown rapidly since then, mainly due to the wide range of actual and proposed applications, which aim at exploiting these materials’ unique medical, mechanical, thermal, electronic and optical properties. In some fields, applications are already a reality, but in most of them, they are the subject of very active research efforts. In photonics, much attention has been paid to the fact that materials with structures with dimensions of a few nanometers fall into the category of metamaterials, since the wavelength of light is longer than the structural scale. This could allow the design of *ex profeso* materials with optical properties for a given application through the manipulation of their structures and compositions at the nanoscale.

This Special Issue of *Micromachines*, entitled ‘Nanomaterials’ Photonics’, presents a collection of articles on different aspects of the use of nanomaterials to implement photonic functionalities. This Special Issue includes a review article entitled ‘Recent Progress in Solution Processed Aluminum and co-Doped ZnO for Transparent Conductive Oxide Applications’ [5], which presents the various efforts made to produce transparent electrical conducting materials, necessary for interfacing different optoelectronic devices.

Regarding the remaining research papers, one, entitled ‘Dual-Criteria Decision Analysis by Multiphotonic Effects in Nanostructured ZnO’ [6], presents a study of the nonlinear optical responses of ZnO nanostructures, which can be useful for encoding and encrypting information. Another article, entitled ‘Volumetric Temperature Mapping Using Light-Sheet Microscopy and Upconversion Fluorescence from Micro- and Nano-Rare Earth Composites’ [7], uses a combination of light-sheet microscopy, a scanning microscopy technique, with a two-dimensional fluorescence intensity ratio to produce 3D temperature maps, employing rare earth-doped nanoparticles. Similarly, the article entitled ‘Tunable Visible Light and Energy Transfer Mechanism in Tm3+ and Silver Nanoclusters within Co-Doped GeO_2_-PbO Glasses’ [8] studies the influence of silver nanoclusters in the emission of light of Tm-doped germanium–lead glasses, as well as the possible applications of these systems for solar cell coatings and novel lights sources in the visible realm. Yet another article, entitled ‘Plasmonic Coupled Modes in a Metal–Dielectric Periodic Nanostructure’ [9], presents a study of the plasmonic properties of a 2D-gap surface plasmon metasurface consisting of gold nanoblocks that present different resonances in the near-infrared context. Finally, the article entitled ‘Theoretical Enhancement of the Goos–Hänchen Shift with a Metasurface Based on Bound States in the Continuum’ [10] proposes the use of metasurfaces exploiting reflection-type bound states in the continuum to achieve very large Goos–Hänchen shifts, which can be useful for sensing devices.

This Special Issue presents a wide range of nanomaterial-related articles in the field of photonics; I hope that they are useful and generate much interest in a constantly evolving field.
